# Single arm meta-analysis of the J-Valve system for aortic regurgitation in Chinese populations

**DOI:** 10.3389/fcvm.2025.1436789

**Published:** 2025-05-21

**Authors:** Lei Gao, Rui Mao, Jie Zeng, Lin Wen

**Affiliations:** ^1^ Department of Medical Equipment, Sichuan Provincial People's Hospital, School of Medicine, University of Electronic Science and Technology of China, Chengdu, China; ^2^ Department of Cardiology, Structure Heart Disease Center, Sichuan Provincial People's Hospital, School of Medicine, University of Electronic Science and Technology of China, Chengdu, China; ^3^ Department of Longquan Campus, Sichuan Provincial People's Hospital, School of Medicine, University of Electronic Science and Technology of China, Chengdu, China

**Keywords:** J-Valve, meta-analysis, aortic regurgitation (AR), Chinese, cardiac valve

## Abstract

**Objective:**

This meta-analysis aimed to evaluate the efficacy and safety of J-valve in patients with Aortic regurgitation (AR).

**Methods:**

PubMed, Embase, the Cochrane Library, and Web of Science databases were searched from inception to November 2024. Primary outcome included Procedural Success, and secondary outcome included In-Hospital Mortality, 30-Day Mortality, One-Year All-Cause Mortality, Stroke Incidence and Complications. The risk of bias was assessed by subgroup analysis, sensitivity analysis, and publication bias, including funnel plot, Egger's test, and Begg's test.

**Results:**

A total of 9 studies involving 552 patients were included in this meta-analysis. The results indicated a surgical success rate of 96% (95% CI: 0.94–0.99). The in-hospital mortality rate was 3% (95% CI: 0.01–0.04), the 30-day mortality rate was 3% (95% CI: 0.01–0.05), and the 1-year all-cause mortality rate was 6% (95% CI: 0.04–0.08). Additionally, the incidence of stroke was 2% (95% CI: 0.01–0.03), and the incidence of other complications was 22% (95% CI: 0.16–0.28).

**Conclusions:**

This meta-analysis indicates that the J-Valve prosthesis exhibits favorable short-term efficacy in patients with severe aortic regurgitation (AR); however, a significant incidence of complications persists. A thorough risk assessment is crucial when determining the appropriate treatment strategy. Furthermore, postoperative follow-up duration should be extended to monitor patient outcomes effectively.

**Systematic Review Registration:**

https://www.crd.york.ac.uk/PROSPERO/view/CRD42024552406, identifier CRD42024552406.

## 1 Introduction

Aortic regurgitation (AR) is characterized by the retrograde flow of blood from the aorta into the left ventricle during diastole (1). This condition can arise from primary diseases of the aortic valve (AV) or from abnormalities in the peri-valvular structures, such as the aortic root and ascending aorta. Globally, AR is the fourth most common type of valvular heart disease (VHD). The Framingham Study estimates the prevalence of AR at 4.9%, with the incidence of moderate to severe AR being 0.5% (2, 3). The incidence and severity of AR increase with age, peaking between 40 and 60 years (4). A recent study on the prevalence of AR in the general rural population of northeastern China found that 4.1% of participants had severe AR (5). Although the prevalence in China is lower than in Western countries, the large population base results in a significant number of individuals requiring treatment for severe AR (6).

Transcatheter aortic valve replacement (TAVR) is the standard treatment for patients with severe symptomatic aortic stenosis (AS) and has also been applied clinically to treat AR. However, the success of TAVR in treating severe AR has been less favorable compared to AS, primarily due to the risk of valve displacement with traditional bioprosthetic valves (7). The J-Valve, a bioprosthetic heart valve independently developed in China, is designed to more effectively treat AR. It features a unique structural design aimed at achieving precise anchoring and reducing the risk of displacement during implantation. While preliminary studies have demonstrated the efficacy of the J-Valve in treating AR, the lack of robust randomized controlled trials has led to ongoing debate regarding its clinical effectiveness and safety (8–11), this limitation has hindered the widespread clinical adoption of the J-Valve Therefore, this study aims to evaluate the efficacy and safety of the J-Valve, providing evidence to support its clinical application.

## 2 Methods

This meta-analysis was conducted based on the preferred reporting items for systematic reviews and meta-analyses (PRISMA) guidelines (12).

### 2.1 Inclusion and exclusion criteria

Inclusion criteria were as follows: (1) Study type: prospective clinical trials and retrospective cohort studies; (2) Diagnosis: patients diagnosed with aortic regurgitation (AR); (3) Intervention: J-Valve prosthetic heart valve; (4) Primary outcome: surgical success rate; (5) Secondary outcomes: in-hospital mortality, 30-day mortality, 1-year all-cause mortality, incidence of stroke, and incidence of complications.

Exclusion criteria were: (1) studies involving fewer than 10 patients; (2) reviews or meta-analyses; (3) non-English publications; (4) studies for which the full text was not available.

### 2.2 Search strategy

A comprehensive search of four databases (PubMed, Embase, Cochrane Library, and Web of Science) was conducted to identify relevant studies assessing the use of the J-Valve. The search period covered from 2015 to November 30, 2024. The following MeSH terms and free-text terms were used in the search: aortic valve insufficiency, insufficiency, aortic valve, aortic regurgitation, regurgitation, aorta, regurgitation, aortic valve, aortic valve insufficiency, insufficiency, aorta, J-Valve. The search strategy used for PubMed was thoroughly adapted for use in the other databases.

### 2.3 Study selection and data extraction

Two reviewers independently screened the articles based on their titles, abstracts, and full texts. Subsequently, the reviewers independently extracted the following data from the included studies: first author, publication year, country, disease, detailed information on the study population and interventions, study design, sample size, and outcomes. Any discrepancies were resolved through discussion. The Newcastle-Ottawa Scale (NOS) was used to assess the risk of bias in non-randomized studies. This scale evaluates the selection of cases and controls, the comparability of cases and controls, and the ascertainment of outcomes. Judgments were made independently by two reviewers, and any differences were resolved through discussion.

### 2.4 Data analysis

All statistical analyses were conducted using STATA 14. Heterogeneity was assessed using the chi-square test and the *I*^2^ statistic, with *P* < 0.1 indicating statistical significance. All data were analyzed using a random-effects model. Additionally, sensitivity analyses were performed to evaluate the stability and reliability of the pooled results. Finally, we assessed potential publication bias.

## 3 Results

### 3.1 Search results and study characteristics

**Figure 1:**
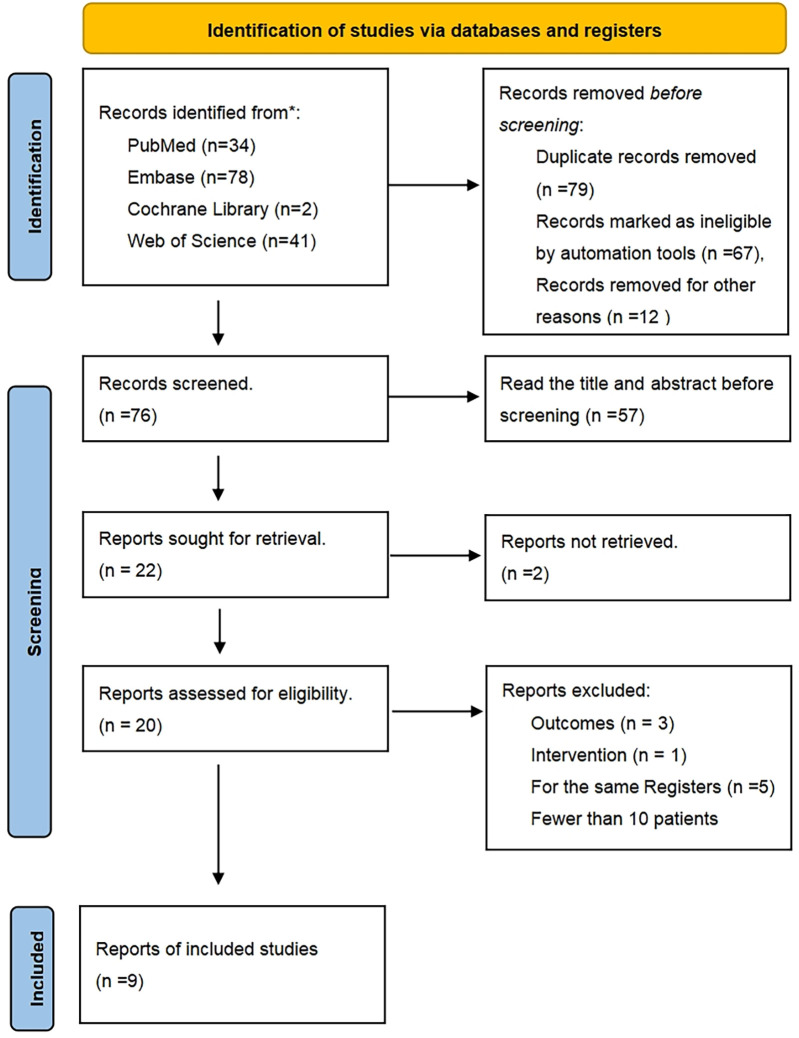
PRISMA flow diagram. Study flow based on the preferred reporting items for systematic review and meta-analysis protocols (PRISMA-P).

The PRISMA flow diagram is presented in Figure 1. After excluding 76 duplicate studies, a total of 155 articles were identified from all databases. Following the screening of titles and abstracts, 22 articles remained. Among these, 2 studies were excluded for not focusing on TAVR, 2 studies were excluded due to differing outcome measures, 5 studies were identified as being from the same research group, 2 studies lacked full-text availability, and 2 studies were excluded due to short follow-up periods and small patient populations. Ultimately, 9 studies, encompassing a total of 552 patients, were included. The screening process is illustrated in Figure 1, and the details of each included study are provided in Table 1a (8, 9, 13–20).

**Table 1a:** Summary of studies.

Author (year)	Study type	Study period	Country	Sample size	Intervention	Mean age	Male	Female
Lu et al. (2022) (13, 14)	Retrospective	2014.5–2019.6	China	27	• J-Valve^TM^	70.6 ± 7.1	29.63%	70.37%
Liu et al. (2022) (20)	Retrospective	2014.3–2019.6	China	134	• J-Valve^TM^	73.1 ± 6.4	25.40%	74.60%
Kong et al. (2022) (9)	Retrospective	2016.9–2021.9	China	69	• J-Valve^TM^	71.46 ± 7.92	24.60%	75.40%
Xue et al. (2021) (15)	Retrospective	2018.1–2019.8	China	22	• J-Valve^TM^	76.5 ± 6.9	55.5%	44.50%
Shi et al. (2021) (8)	Prospective	2014–2016	China	107	• J-Valve^TM^	77.1 ± 5.4	48.6%	51.40%
Liu et al. (2020) (16)	Retrospective	2014.5–2018.10	China	47	• J-Valve^TM^	73.7 ± 7.9	72.3%	27.70%
Li et al. 2020 (17)	Prospective	2014.7–2015.6	China	18	• J-Valve^TM^	74.50 ± 5.22	27.8%	72.20%
Tung et al. (2018) (18)	Prospective	2014.3–2015.7	China	107	• J-Valve^TM^	74.4 ± 5.2	45.8%	54.20%
Luo et al. (2017) (19)	Prospective	2014.7–2015.6	China	21	• J-Valve^TM^	75.52 ± 5.22	66.7%	33.30%

Table 1b summarizes the characteristics of the patient cohort. The mean age of the patients was 74.10 ± 6.36 years, with more than half being male (63.41%). Three cardiac risk assessment models were utilized across the studies: the Society of Thoracic Surgeons (STS) model, which is a widely accepted scoring system that integrates numerous clinical variables such as age, gender, weight, and medical history to accurately quantify the risk level for each patient undergoing specific cardiac surgeries, categorizing them into low, intermediate, and high-risk groups; the European System for Cardiac Operative Risk Evaluation (Log EuroSCORE), which primarily assesses the overall risk of cardiac surgery, including valve surgeries, coronary artery bypass grafting (CABG), and considers perioperative mortality and other complications; and the mean left ventricular ejection fraction (LVEF), a crucial parameter for evaluating the risk suitability for AR surgery.

**Table 1b:** Patient characteristics.

Characteristics	No. of publications with data	Overall no. of patients	No. of events	Weighted mean
Age (years)	9	552	N/A	74.12 ± 6.26
Male gender (%)	9	552	350	63.41 ± 13.37
STS score (%)	4	246	N/A	8.67 ± 4.11
Logistic EuroSCORE (%)	4	202	N/A	24.93 ± 8.15
Logistic EuroSCORE II (%)	5	302	N/A	17.62 ± 5.72
NYHA 1 and 2 (%)	5	397	N/A	6.73 ± 1.96
NYHA 3 and 4 (%)	9	552	N/A	87.47 ± 8.51
CAD (%)	2	134	26	19.40 ± 3.53
Previous CABG (%)	5	327	28	8.56 ± 0.75
PAD (%)	4	203	82	40.39 ± 19.06
COPD (%)	5	221	90	40.88 ± 21.1
DM (%)	5	221	32	14.48 ± 8.94
LVEF (%)	8	505	N/A	55.15 ± 9.59
Hypertension (%)	4	114	75	65.79 ± 15.38
AF (%)	7	376	83	22.07 ± 5.41
CKD (%)	2	49	10	20.41 ± 4.89

Except for one study, all studies reported the mean LVEF, with an average value of 55.15 ± 9.59%. All studies included patients classified as New York Heart Association (NYHA) functional class III/IV, with over three-quarters (87.47 ± 8.51%) experiencing heart failure. Table 1a also summarizes the prevalence of cardiovascular comorbidities such as coronary artery disease (CAD), peripheral arterial disease (PAD), and a history of coronary artery bypass grafting (CABG).

Except for two studies, all studies reported the prevalence of atrial fibrillation (AF), with an average prevalence of 22.07 ± 5.41%. Additionally, most studies reported chronic obstructive pulmonary disease (COPD) and diabetes mellitus (DM). Four studies reported on hypertension and peripheral vascular disease (PVD), while two studies reported on coronary artery disease (CAD) and chronic kidney disease (CKD). Hypertension had a particularly notable average prevalence of 65.79%. Only one study reported dyslipidemia, anemia, and pulmonary hypertension, with prevalences of 14.9%, 38.3%, and 33.6%, respectively.

### 3.2 Quality of the included studies

Two non-randomized studies were evaluated using the Newcastle-Ottawa Scale (NOS), which assesses studies based on eight items grouped into three domains: selection of the study groups, comparability of the groups, and outcome (for cohort studies) or exposure (for case-control studies) assessment. The quality assessment details are shown in Table 2.

**Table 2:** Quality assessment of the studies included in the meta-analysis.

Study/year	Q1	Q2	Q3	Q4	Q5	Q6	Q7	Q8	Total
Lu et al. (2022) (13, 14)	1	0	1	1	0	1	1	1	6
Liu et al. (2022) (20)	1	0	1	1	0	1	1	1	6
Kong et al. (2022) (9)	1	1	1	1	1	1	0	1	7
Xue et al. (2021) (15)	1	0	1	1	0	1	1	1	6
Shi et al. (2021) (8)	1	0	1	1	0	1	1	1	6
Liu et al. (2020) (16)	1	0	1	1	0	1	1	1	6
Li et al. (2020) (17)	1	0	1	1	0	1	1	1	6
Tung et al. (2018) (18)	1	0	1	1	0	1	1	1	6
Luo et al. (2017) (19)	1	0	1	1	0	1	1	0	5

Newcastle–Ottawa Scale (NOS) for included non-randomized studies.

Numbers Q1–Q8 in heading signified: Q1, were there clear criteria for inclusion in the case series? Q2, Selection of the non-exposed cohort; Q3, were valid methods used for identification of the condition for all participants included in the case series? Q4, Demonstration that outcome of interest was not present at start of study; Q5, Comparability of cohorts based on the design or analysis; Q6, Assessment of whether the results are accurate or continuous; Q7, was follow-up long enough for outcomes to occur? Q8, were the outcomes or follow-up results of cases clearly reported?

### 3.3 Procedural success

**Figure 2:**
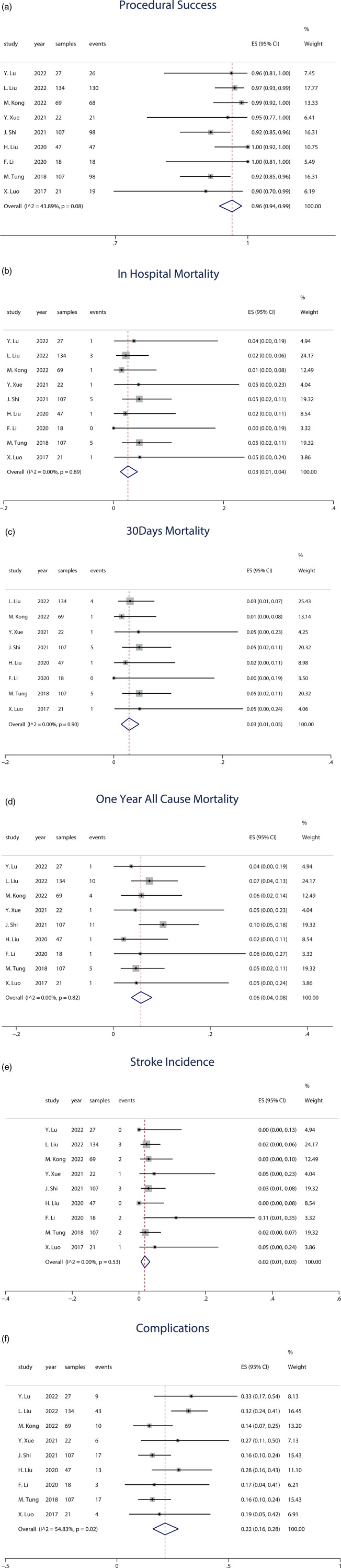
**(a)** Forest plot of procedural success. **(b)** Forest plot of in-hospital mortality. **(c)** Forest plot of 30-day mortality. **(d)** Forest plot of 1-year all-cause mortality. **(e)** Forest plot of 1-year stroke incidence. **(f)** Forest plot of 1-year stroke incidence.

All studies reported the procedural success rate of J-Valve implantation. The results demonstrated an overall success rate of 96% (95% CI: 0.94–0.99). In terms of the number of successfully performed procedures, all cases in the studies by Liu et al. (16) and Li et al. (17) were successful, while Liu et al. (20) reported 4 failed cases, and Shi et al. (8) and Tung et al. (18) each reported 9 failed cases, indicating significant absolute heterogeneity. Overall, the heterogeneity in procedural success rates was relatively low (*I*^2^ = 43.89%). The forest plot in Figure 2a illustrates the results of all studies. The forest plot depicted in Figure 2a exhibits the outcomes of all investigations.

### 3.4 In-hospital mortality

All studies included data on in-hospital mortality following J-Valve implantation, with a total sample size of 552 patients. The analysis revealed an overall in-hospital mortality rate of 3% (95% CI: 0.01–0.04). The forest plot in Figure 2b presents the results from all studies.

### 3.5 30-day mortality

Among the 9 studies, 8 provided data on 30-day mortality, encompassing a total of 525 patients, with 18 deaths occurring within 30 days post-procedure. The weighted average 30-day mortality rate was 3% (95% CI: 0.01–0.05). Three studies with smaller sample sizes, ranging from 18 to 22 patients, reported varying outcomes: one study reported a 30-day mortality of 0, while the other two studies each reported 1 death. The forest plot in Figure 2c illustrates the results of the reported studies.

### 3.6 One-year all-cause mortality

The weighted average across all studies was 6% (95% CI: 0.04–0.08). Among the 9 studies, 8 reported data on 1-year all-cause mortality, involving a total of 445 patients, with 24 deaths. Additionally, one study reported 2-year all-cause mortality, including 107 patients, with 11 deaths. The forest plot in Figure 2d presents the results from all studies.

### 3.7 Stroke incidence

All included studies reported stroke events as an outcome. Among the 9 studies, 2 reported no occurrence of stroke, while 7 studies documented stroke incidence, involving a total of 478 patients and 14 stroke events. The weighted average incidence of stroke was 2% (95% CI: 0.01–0.03). The forest plot in Figure 2e illustrates the results from the studies.

### 3.8 Complications

In the random-effects model (*I*^2^ = 54.83%, *p* = 0.02), the pooled complication rate was 22% (95% CI: 0.16–0.28). The forest plot in Figure 2f illustrates the results of the studies. Table 3 provides an overview of the acute procedural complications reported as outcomes in the included studies.

**Table 3:** Acute procedural complications.

Complications	No. of studies	Overall no. of patients	No. of events	ES (CI 95%)	Weighted mean (%)
PVL	16, 21, 29, 33, 45	272	91	0.33 (0.28, 0.39)	2.28 ± 0.33
Permanent pacemaker insertion	16, 18, 21, 45, 61, 73	405	27	0.07 (0.05, 0.10)	12.15 ± 0.07
Major bleeding	16, 18, 21, 45, 61, 73	405	19	0.05 (0.03, 0.07)	16.91 ± 0.05
Acute kidney injury	16, 18, 45, 61	315	16	0.05 (0.03, 0.08)	12.20 ± 0.05
Valve-related reintervention	16, 18, 33, 45	315	8	0.03 (0.01, 0.05)	23.76 ± 0.03
Valve thrombosis	16, 18, 45, 46, 61	333	11	0.03 (0.02, 0.06)	19.46 ± 0.03
Other		552	47	0.09 (0.06, 0.11)	13.23 ± 0.09

A total of 5 studies involving 272 patients reported 91 cases of perivalvular aortic regurgitation (PAR), with an incidence rate of 33% and a weighted mean of 2.28 ± 0.33. The highest incidence was reported by J. Shi in (8), with 57% (91/107) of cases. M. Kong's (9) study also reported 23 cases (23/69). Six studies (involving 405 patients) reported the need for permanent pacemaker insertion (PPI), with an incidence rate of 6.7% and a weighted mean of 12.15 ± 0.07. Liu's et al. (20) study reported 12 cases (12/134).

Among the 6 studies, 19 cases of major bleeding were reported, with a weighted mean of 16.91 ± 0.05. Of these, 3 cases required blood transfusions, 1 case required intraoperative hemostasis, and 10 cases involved postoperative bleeding.

Additionally, 4 of the 9 studies reported 16 cases of acute kidney injury (AKI) out of 315 patients, with a weighted mean of 12.20 ± 0.05. Eight cases required valve-related reintervention, with a weighted mean of 23.76 ± 0.03, making it the most significant factor among complications. Furthermore, 5 of the 9 studies reported 10 cases of valve thrombosis (VT) out of 315 patients, with a weighted mean of 19.46 ± 0.03. Other complications had a weighted mean of 13.23 ± 0.09.

Liu's et al. (20) study reported 9 cases of intraoperative coronary artery obstruction (CAO), 2 cases of cardiac tamponade (CT), 1 case of endocarditis, and 11 cases of arrhythmia. Xue's et al. (15) study reported 2 cases of postoperative new-onset conduction block and 1 case of postoperative left ventricular pseudoaneurysm (LVP). Shi's et al. (8) study reported 6 cases of third-degree atrioventricular block (AVB) and 5 cases that required conversion to open-heart surgery. Li's et al. (17) study reported 1 case of myocardial infarction (MI) and 1 case of paroxysmal atrial fibrillation (PAF). Tung's et al. (18) study reported 6 cases of conversion to open-heart surgery and 1 case of arteriovenous fistula (AVF).

### 3.9 Sensitivity analysis

**Figure 3:**
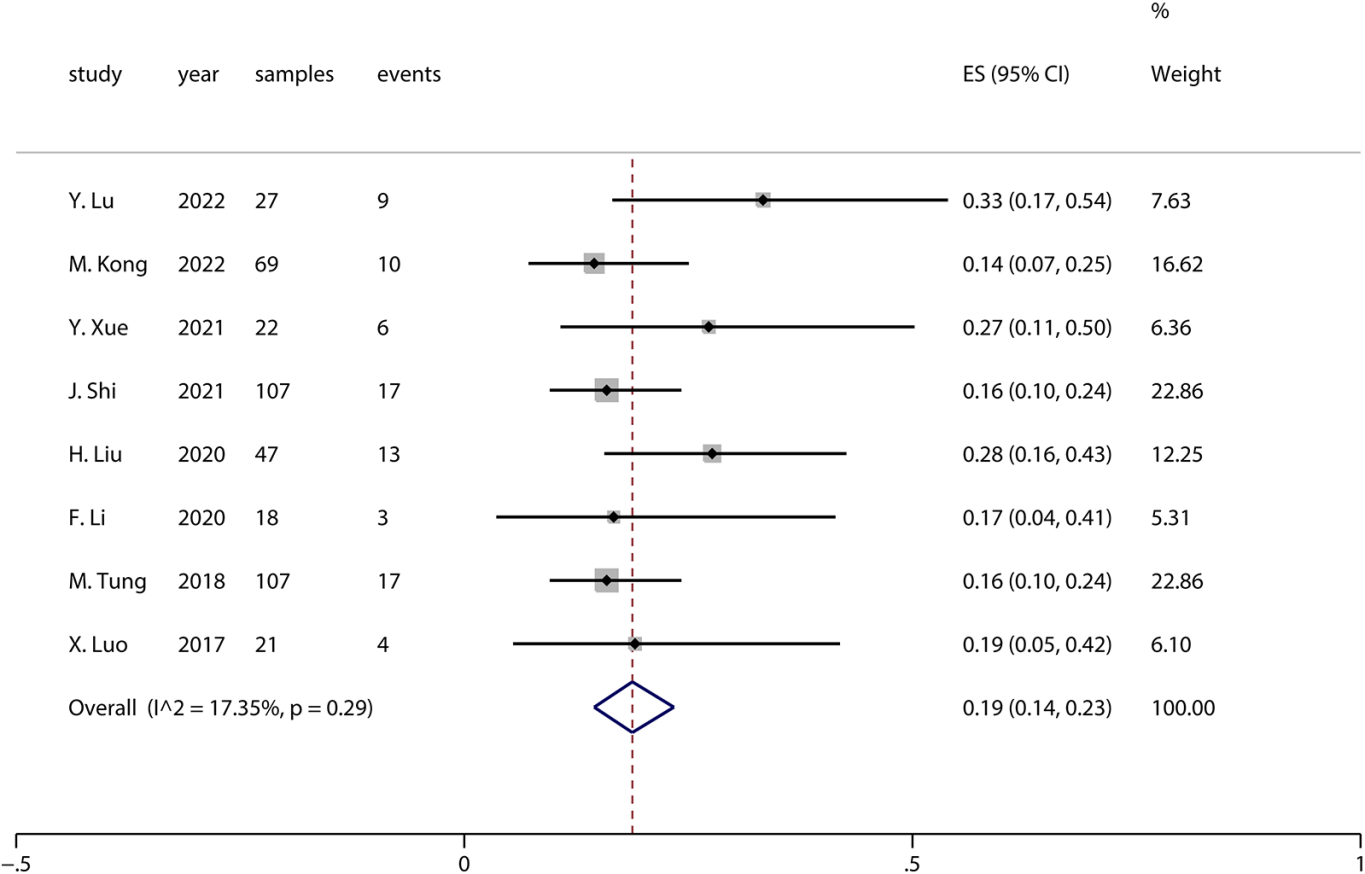
Complications after sensitivity analysis.

Sensitivity analysis is a method used to evaluate the impact of a specific variable on the overall outcome of a study by individually excluding that variable and reanalyzing the data. In this study, moderate-to-high heterogeneity was observed when pooling the effect sizes for complications (*I*^2^ = 54.83%, *p* = 0.02). The analysis revealed that the study by Liu et al. in (20) had a significant impact on the pooled complication results. Upon excluding this study, the pooled complication rate across all studies was 0.19 (*I*^2^ = 17.35%, *p* = 0.29), as shown in the forest plot in Figure 3. The forest plot, excluding Liu et al. (20), indicates that the complication rate was 0.19 (95% CI: 0.14–0.23), which is slightly lower than the previously pooled rate of 0.22 (95% CI: 0.16–0.28), though not significantly so. This suggests that the results of this meta-analysis are generally reliable.

### 3.10 Publication bias

**Figure 4:**
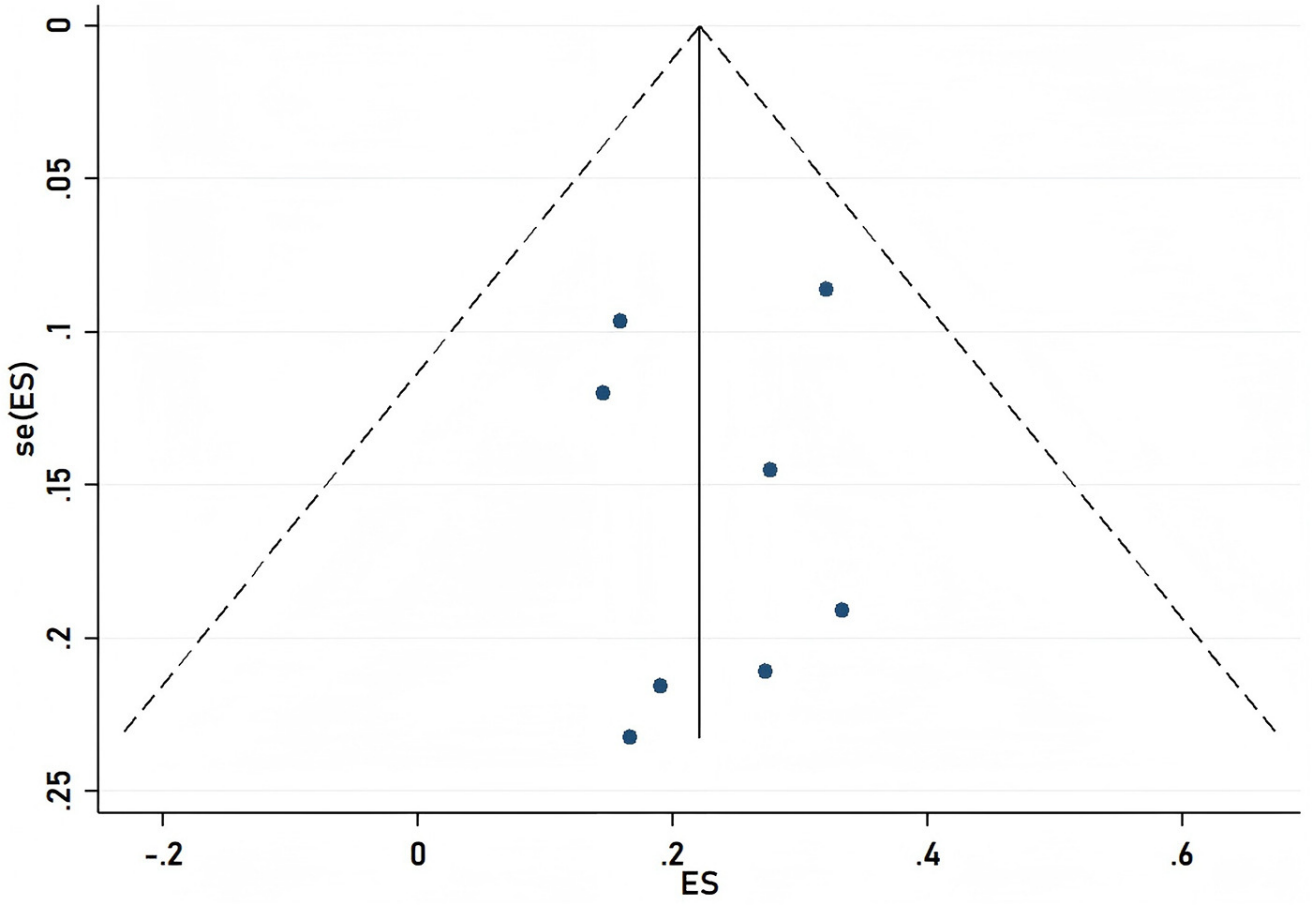
Funnel plot funnel with pseudo 95% confidence limits.

To ensure the validity of the meta-analysis results, we employed a funnel plot for observation and used the propensity score matching (PSM) method to estimate the likelihood of publication bias. The PSM method estimates the potential propensity scores of the treatment group and matches them to reduce differences between the two groups prior to treatment, thereby mitigating the effects of selection bias. Upon examination, the funnel plot displayed a symmetrical shape, and the *P*-values from the PSM method were all greater than 0.05, indicating no evidence of publication bias as shown in Figure 4.

## 4 Discussion

For high-risk patients who are not candidates for traditional surgery, TAVR has rapidly become a primary option for treating aortic valve-related diseases (7). In the past, bioprosthetic valves used in TAVR for treating AR were often used off-label, resulting in lower surgical success rates; even when successfully implanted, there was a higher rate of complications (21–23). This is a significant reason why only about 5% of AR patients have undergone TAVR (24). For example, severe aortic root or annular dilation, infective endocarditis (IE), aortic dissection (AD), and valve tumors leading to aortic regurgitation (AR) are absolute contraindications for transcatheter aortic valve replacement (TAVR). However, when AR is caused by leaflet failure, TAVR may still be considered. The primary challenge, in this case, is that the lack of valve calcification makes it difficult for the bioprosthetic valve to anchor securely, leading to potential valve dislodgement or migration. This makes TAVR a significant clinical challenge in the treatment of AR (25). Recent studies indicate that the complication rate for TAVR with bioprosthetic valves within the indications is lower compared to off-label uses (26, 27). The J-Valve has been approved by the China Food and Drug Administration (CFDA), with AR explicitly included within its indications. The outcomes in this study show a surgical success rate of 96% (95% CI 0.94–0.99) and an in-hospital mortality rate of 3% (95% CI 0.01–0.04), with a 30-day average mortality rate of 3% (95% CI 0.01–0.05), indicating its advantages in treating AR compared to other off-label valve uses.

There was no significant difference between 30-Day Mortality and In-Hospital Mortality. In the included studies, the number of perioperative deaths was consistent with the 30-day mortality count, with only one additional death reported in the 30-day mortality in Liu's et al. (20) study. However, there was a significant difference between 30-Day Mortality and One-Year All-Cause Mortality [30-Day Mortality was 3% (95% CI 0.01–0.05), 1-year all-cause mortality rate was 6% (95% CI 0.04–0.08)]. Although the data from 30 days and 1 year show a rising trend in 1-year mortality, this level remains relatively low compared to recent related studies (28–30). This suggests that the J-Valve has a favorable short-term effect, though there may still be a trend of increasing mortality risk over the long term, yet it remains superior to traditional treatment methods.

**Figure 5:**
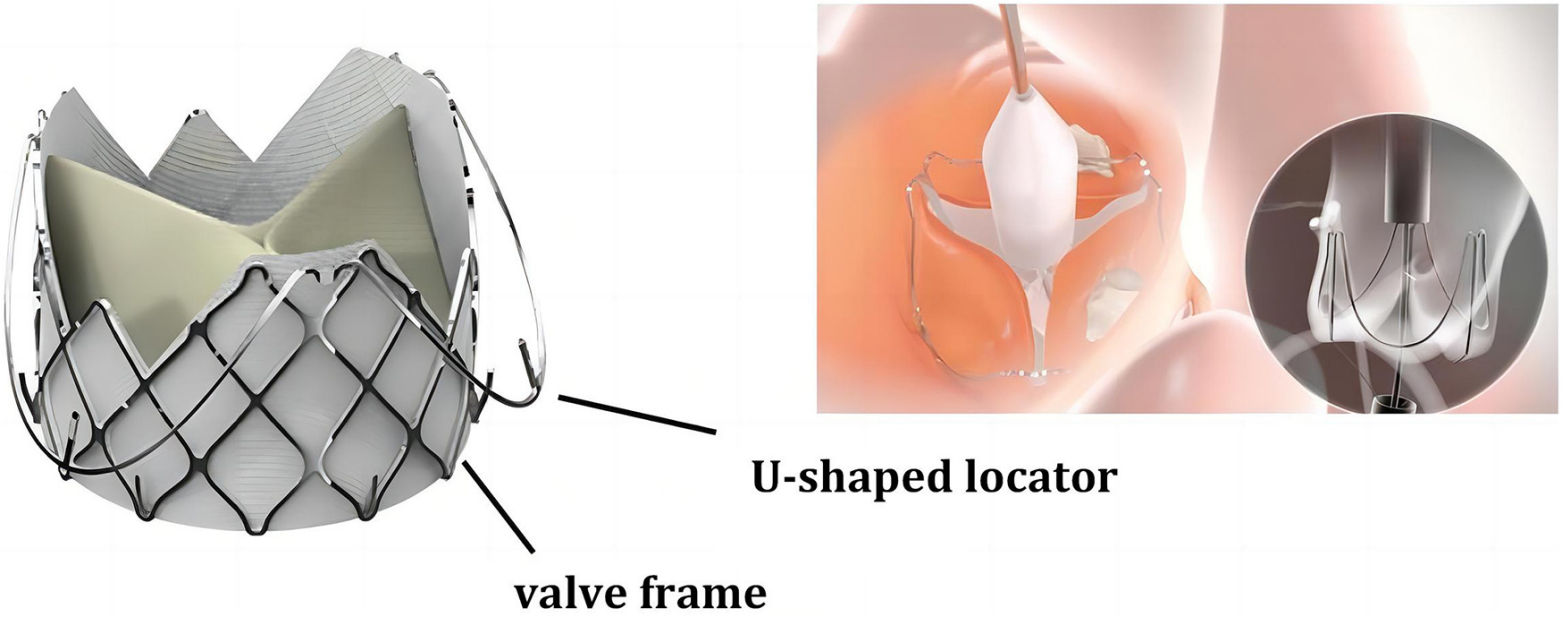
J-valve schematic diagram of structure.

AR is often accompanied by annular and ascending aortic dilatation, and the accuracy and stability of Transcatheter Heart Valve (THV) positioning significantly impact the incidence of complications such as Paravalvular Leak (PVL), conduction disturbances, or annular rupture. Generally, prosthesis anchoring mainly depends on the oversized radial force of the THV, but excessive valve dilation can worsen complications like PVL. Therefore, a lack of anchoring and the presence of migration remain major issues with THV. The J-Valve consists of a nitinol stent attached to the aortic leaflets and three U-shaped positioning holders surrounding the stent, which assist in valve positioning during deployment and enhance anchoring (as shown in Figure 5). This design allows the surgeon to place the valve in the ideal position more easily, without the need for rapid ventricular pacing, thereby avoiding the need for multiple position adjustments during traditional valve implantation, simplifying the surgical procedure, and contributing to the higher surgical success rate. Precise positioning also means reducing the occurrence of postoperative complications such as PVL. Since the J-Valve can be more accurately anchored within the aortic sinus, it reduces the risk of valve displacement or misalignment leading to PVL, which not only improves surgical success rates but also lessens the postoperative recovery burden on patients. The unique design of the J-Valve system simplifies the positioning and adjustment process, shortens surgery time, reduces anesthesia duration, and lowers surgical risks. Moreover, in cases of valve dilation regurgitation, compared to other types of valves, the J-Valve significantly reduces the risk of valve slippage into the ventricle.

For valve diseases such as, patients often have valve calcification, whereas AR patients do not have calcified valves. Other valves typically have skirts that are easier to fix to calcified valves. In contrast, the J-Valve does not have a skirt. For AR patients, the J-Valve embraces the native leaflets through its positioning holders, naturally forming a skirt. This naturally formed skirt also positively affects PVL resulting from later valve dilation. In the included literature, at least 91 cases of PVL were reported, with 77 cases of mild PVL [M. Tung et al. (18), did not provide specific data, but indicated that all patients had mild or less severe PVL during 1-year follow-up], 14 cases of moderate PVL, and no cases of severe PVL. The occurrence of PVL at varying degrees is closely related to the patient's anatomical structure, valve implantation conditions, and surgical procedures. These factors interact, leading to differences in the severity of PVL. Abnormalities in the patient's anatomical structure, such as a bicuspid aortic valve, may prevent perfect valve fit after implantation, thereby increasing the risk of mild PVL. Inaccurate valve positioning during implantation can result in small gaps between the valve and the annulus, leading to mild PVL. Selection of an incorrect valve size, especially a valve that is relatively small compared to the aortic annulus, may fail to fully seal the annular space, resulting in mild PVL. Excessive expansion of the aortic annulus beyond the valve's adaptation range can hinder proper sealing of the annular gap, even if the valve is successfully implanted, leading to moderate PVL. Surgical manipulation may cause some degree of valve damage, affecting its normal function and potentially leading to moderate PVL. Severe PVL typically results from significant valve displacement, causing a large gap around the annulus. Severe damage to the valve structure during surgery can lead to the loss of its normal sealing function. Severe damage to surrounding tissues during surgery can lead to significant defects around the annulus, causing severe PVL. However, severe PVL is relatively rare in the application of the J-Valve system based on the overall study.

Previous studies have shown that PVL negatively impacts both short-term and long-term survival in AR patients (31, 32). The incidence of PVL in similar studies varies from 7% to 40%, whereas the incidence of PVL in this study is much lower (33, 34). Thus, the J-Valve, even without a skirt, still has advantages in treating AR. Furthermore, the positioning advantage of the J-Valve can effectively assist less experienced surgeons, and the simplified procedure aids in the widespread dissemination and application of the technique.

The incidence of Acute Kidney Injury (AKI) ranges from 8.3% to 58% (35). A recent study involving 366 patients reported an incidence of 14.5% of AKI following TAVI (36). In this study, the incidence of AKI post-TAVI was only 5% (95% CI 0.03–0.08). The overall incidence of stroke within 30 days is 3%–4%, while the combined incidence in this study was only 2% (95% CI 0.01–0.03) (37). Almarzooq's study pointed out that TAVI-related stroke is associated with a higher risk of 1-year composite outcomes (38). The results of this study, combined with Almarzooq's findings, indirectly reflect that the J-Valve reduces the incidence of stroke post-implantation. Although complications such as valve embolism and migration may occur during surgery, leading to increased complications and mortality, these events were not observed in the studies included in this paper (39). After J-Valve implantation, patients showed significant improvements in echocardiographic indicators, NYHA classification, left ventricular ejection fraction (LVEF), and left ventricular size, significantly enhancing safety and effectiveness. These conclusions are consistent with recent research findings (14, 40, 41).

Nine studies have examined the use of pacemaker therapy in the treatment of aortic valve disease with the J-Valve. The sample sizes and follow-up durations varied across these studies. Overall, the pacemaker implantation rate showed variability (as detailed in Table 3). This variability is primarily related to factors such as the patient's intrinsic cardiac conduction system condition, the impact of valve implantation on the conduction system, and the design and implantation depth of the valve. For instance, in Lu et al. (13, 14) among 27 patients, 1 (3.7%) required pacemaker therapy after 9 months due to complete atrioventricular block. In “Liu et al.” (20) 12 out of 134 patients required implantation of a new permanent pacemaker. Some studies also mentioned the timing of pacemaker implantation. For example, 10 cases required implantation within 30 days postoperatively, and 2 cases were implanted during follow-up. In “Shi et al.” (8) 4 cases developed third-degree atrioventricular block during a 30-day follow-up. The patient's intrinsic cardiac conduction system condition is a critical factor. Some patients may have pre-existing conduction system issues, such as varying degrees of atrioventricular block, which increases the likelihood of requiring pacemaker therapy following J-Valve implantation. The implantation procedure may impact the cardiac conduction system. Precision during the procedure is crucial; damage to surrounding tissues, especially near the conduction bundle, during J-Valve implantation may cause conduction block, necessitating pacemaker therapy. Factors such as the design and implantation depth of the J-Valve are also related to the need for pacemaker therapy. Although the unique structure and anchoring mechanism of the J-Valve offer advantages, excessive implantation depth may compress or interfere with the cardiac conduction system, increasing the need for pacemaker implantation. Pacemaker therapy, being an invasive procedure, carries inherent risks. Complications such as infection and bleeding may arise during surgery, which can not only affect postoperative recovery and prolong hospital stay but also interfere with the therapeutic effect of the J-Valve. For example, infection may lead to peri-valvular tissue inflammation, affecting the valve's stability and function, which in turn impacts the therapeutic effect of the J-Valve on aortic valve disease. Pacemaker therapy increases both the psychological burden and the economic cost for patients. Patients must undergo implantation of a new medical device and subsequent follow-up management, which may affect their quality of life and treatment adherence. If patients are unable to attend follow-up visits or adhere to treatment protocols due to psychological stress or financial constraints, it may indirectly affect the long-term therapeutic outcomes of the J-Valve. The study also mentioned that, compared to other valves, the pacemaker implantation rate for the J-Valve is relatively low. This may be due to its design, which somewhat reduces adverse effects on the conduction system, though it cannot eliminate such occurrences.

The bleeding and transfusion rates varied across the nine studies, with differences observed between the studies. Some studies reported a low incidence of bleeding, while others reported a higher incidence (as detailed in Table 4). The occurrence of bleeding is associated with the surgical procedures involved in J-Valve application, the patient's underlying condition, and the classification of bleeding events based on the BARC grading system. The invasive nature of the surgical procedures involved in J-Valve application is one of the primary causes of bleeding. During the implantation of the J-Valve, both catheter-based procedures and other related surgical steps may cause damage to blood vessels, cardiac tissues, and other structures. For example, during puncture procedures, the vessel wall may be damaged, leading to bleeding. The complications at the puncture site mentioned in “Liu et al.” (20) may have been caused by improper puncture technique. The patient's underlying health condition also influences the occurrence of bleeding. Some patients may have coagulopathy or other underlying conditions that increase the risk of bleeding. In “Lu et al.” (13, 14) a patient received a transfusion preoperatively due to anemia. Although this was not due to major intraoperative bleeding, it highlights how the patient's blood condition may predispose them to bleeding during surgery. The classification of bleeding events based on the BARC grading system can be influenced by various factors related to J-Valve application. During surgery, even seemingly minor bleeding events, if prolonged or if the cumulative bleeding volume reaches a certain threshold, may be classified as more severe bleeding events. Following J-Valve implantation, if a patient requires multiple transfusions, is hospitalized due to bleeding, or undergoes surgical intervention to control bleeding, these events would be classified as severe bleeding events according to the BARC grading system. This demonstrates how various factors in the J-Valve application process can influence the classification of bleeding events, underscoring the need for a comprehensive assessment of multiple factors when evaluating the efficacy of J-Valve.

**Table 4:** Report of bleeding academic research consortium (BARC) classification.

Author (year)	No. of events	BARC
Lu et al. 2022.pdf (13, 14)	1	1
Liu et al. 2022.pdf (20)	1	3
Kong et al. 2022.pdf (9)	5	3
Xue et al. 2021.pdf (15)	1	3
Shi et al. 2021.pdf (8)	0	\
Liu et al. 2020.pdf (16)	2	2
Li et al. 2020.pdf (17)	0	\
Tung et al. 2018.pdf (18)	1	3
Luo et al. 2017.pdf (19)	1	3

The reasons for patients requiring valve-related reintervention are diverse, with the most common being prosthetic valve thrombosis, valve displacement, and paravalvular leakage. Prosthetic valve thrombosis is one of the more common causes. Thrombosis severely affects valve function, obstructs smooth blood flow, and leads to hemodynamic abnormalities. This may be related to factors such as postoperative anticoagulation compliance, individual hypercoagulability, and the interaction between the valve material and blood. Valve displacement is another significant cause (42). In one case, valve displacement occurred to the aortic arch during surgery. This typically happens during valve implantation, possibly due to inaccurate positioning, improper placement, or anatomical mismatch, which leads to displacement under the blood flow pressure from the beating heart. Only in Xue et al. (15), cases of valve displacement during surgery required reintervention in the acute phase. In the subacute phase, reoperation due to prosthetic valve thrombosis was performed in Liu et al. (20) and Shi et al. (16). Other cases involved long-term reintervention after implantation. Over time, the heart requires greater effort to pump blood through the obstructed valve, potentially leading to complications such as heart failure. Reintervention in the acute or subacute phase restores normal blood flow and prevents further cardiac damage from ischemia or hypoxia. Other cases reported long-term reinterventions, but specific details were not provided. The outcomes of these reinterventions are notably uncertain. Successful reintervention can effectively resolve valve-related issues and significantly improve cardiac function and quality of life.

The included studies analyzed the risk of patients undergoing cardiac surgery using two risk assessment models: the widely accepted Society of Thoracic Surgeons (STS) score and the Log European System for Cardiac Operative Risk Evaluation (EuroSCORE). However, from the included studies, it was found that doctors in China prefer to use NYHA and LVEF to assess whether patients require intervention with the J-Valve. Preliminary evidence from the studies included in this paper suggests that following J-Valve treatment for AR, most patients experienced symptom relief at one-year follow-up, with NYHA decreasing from III–IV to I–II, significant improvement in LVEF scores, and a marked improvement in left ventricular geometry.

AR is more common in men than in women, possibly due to the higher prevalence of bicuspid aortic valve disease in men (43). Cardiac remodeling is caused by volume and combined pressure-volume overload, which appears to develop differently in men and women. For example, in cardiac MRI studies, left ventricular (LV) dilation in men is closely associated with AR regurgitation fraction, whereas women differ from men in this regard. LV dilation occurs more frequently in men compared to women (91.3% vs. 64.7%). Regarding the impact of gender on In-Hospital Mortality, studies have shown that women have significantly higher perioperative mortality rates than men (44). Although current valvular heart disease guidelines recommend triggering intervention based on specific thresholds of LV systolic dysfunction and dilation, they do not differentiate between men and women. This undifferentiated approach may lead to significant differences in the referral for aortic valve surgery for severe AR between men and women, impacting treatment outcomes. In the studies included in this paper, women accounted for an average of 36.59%, but none mentioned the impact of gender on J-Valve implantation, warranting further in-depth research.

Analyzing these studies reveals that patient-related factors, surgical procedures, valve-related issues, and postoperative management can all contribute to surgical failure. Multiple studies indicate that patients with comorbidities such as hypertension, diabetes, and chronic obstructive pulmonary disease (COPD) are at higher surgical risk. In Liu et al. (20), patients often had multiple underlying conditions, which increased surgical complexity and risk, potentially leading to failure. Patients with poor cardiac function, such as those with low left ventricular ejection fraction (LVEF), have poorer surgical tolerance. Lu et al. (13, 14) studied patients with aortic valve regurgitation combined with left ventricular dysfunction. These patients, already having poor cardiac function, are more prone to complications during surgery, affecting the outcomes and potentially leading to failure. The accuracy and standardization of surgical procedures are crucial for successful outcomes. Incorrect valve placement is a critical issue. In Xue et al. (15), one patient required a second implantation due to valve displacement, highlighting that improper positioning can affect valve function and lead to surgical failure. Damage to surrounding tissues during surgery can have severe consequences. For example, vascular injury during puncture can lead to major bleeding and other complications, disrupting the procedure and potentially resulting in surgical failure. The learning curve of surgical procedures should not be overlooked. Inexperienced surgeons are more prone to errors, increasing the likelihood of failure. Valve selection and quality directly impact surgical outcomes. An improper valve size can cause numerous issues. Liu et al. (16) reported that mismatched valve size and aortic annulus may lead to paravalvular leakage (PVL), affecting cardiac function and potentially causing surgical failure. The structure and performance of the valve are also critical. If the valve has quality issues or design defects, postoperative dysfunction such as thrombosis or leaflet malfunctions may occur, leading to surgical failure. Postoperative complications are a major cause of surgical failure. Paravalvular leakage (PVL) is mentioned in several studies, such as Lu et al. (13, 14) and Liu et al. (20). Moderate to severe PVL disrupts normal hemodynamics, leading to cardiac dysfunction and, in severe cases, surgical failure. Prosthetic valve thrombosis is another serious issue. Patients in Liu et al. (20) and Shi et al. (8) required reintervention due to thrombosis, indicating that thrombosis disrupts valve function, affecting surgical outcomes and potentially causing failure. Arrhythmias, such as third-degree atrioventricular block, require pacemaker therapy and may affect cardiac function, thereby influencing surgical outcomes.

In terms of structural stability and durability, only one study reported that the J-Valve prosthesis maintained good structural stability over a period of at least 4 years. Its components, including the stent and leaflets, showed no significant damage, deformation, or degradation, thus preserving normal valve function. In other studies, although such long-term morphological evaluations were not conducted, no severe valve structural issues were observed during relatively shorter follow-up periods, indirectly supporting the potential for long-term structural stability. From a materials science perspective, the nickel-titanium alloy stent and porcine aortic valve tissue of the J-Valve prosthesis can resist fatigue, wear, and corrosion over the long term in the human body, ensuring its durability. This structural stability and durability are fundamental to its long-term biocompatibility, ensuring that the valve functions effectively over time and reducing the need for reinterventions or adverse events due to structural issues. None of the studies reported severe hemodynamic disturbances, indicating that the J-Valve prosthesis maintains good hemodynamic performance during long-term use, allowing the heart to function in a relatively normal flow state. An appropriate transvalvular pressure gradient ensures the heart's pumping efficiency, reducing cardiac workload and preventing heart dysfunction caused by hemodynamic abnormalities. Good hemodynamic compatibility also reduces turbulence and vortices around the valve, lowering the risk of thrombosis and further ensuring the valve's long-term biocompatibility. During the follow-up in various studies, no valve dysfunction or increase in adverse events due to severe tissue inflammation was observed. This suggests that the J-Valve prosthesis does not trigger excessive immune or inflammatory responses after long-term implantation, and the body's tissues adapt well to the presence of the valve. The J-Valve prosthesis has minimal impact on the overall body, causing little damage to the function of other vital organs such as the liver and kidneys. Follow-up in various studies did not report any deterioration in liver or kidney function due to valve implantation, indicating good overall biocompatibility between the valve and the body, without adverse systemic effects.

## 5 Limitation

The limitations of this study include the small sample sizes of most studies, which may not adequately represent the diversity of patient conditions. The short follow-up periods also make it difficult to assess long-term outcomes. Additionally, many studies are retrospective in design and lack prospective randomized controlled trials, making them susceptible to bias and limiting the accurate assessment of their advantages. Variations in surgical procedures, postoperative management, and outcome measure assessments across studies further complicate the meta-analysis. Future research should include long-term follow-up studies and randomized controlled trials.

## 6 Conclusions

This article explores the safety and efficacy of the J-Valve cardiac valve in the treatment of AR. The study indicates that the J-Valve has advantages in addressing non-calcified aortic regurgitation. It boasts a high surgical success rate, significant post-operative improvement in cardiac function, and a low incidence of complications. The unique design of the J-Valve facilitates precise implantation and reduces PVL. These findings support the J-Valve as a viable alternative therapy for high-risk patients and provide scientific evidence for its broader future application. However, given that other devices have shown more promising data, I believe that this valve may not be the most suitable option for treating aortic valve regurgitation.

## Data Availability

The original contributions presented in the study are included in the article/Supplementary Material, further inquiries can be directed to the corresponding author.
